# Liver Function Test Results Correlate With Spleen Size in Patients With Infectious Mononucleosis

**DOI:** 10.7759/cureus.70041

**Published:** 2024-09-23

**Authors:** Maxwell S Durtschi, Nicole S Pham, Calvin E Hwang

**Affiliations:** 1 Department of Orthopaedic Surgery, Stanford University School of Medicine, Stanford, USA

**Keywords:** ebv splenomegaly, infectious mononucleosis (im), return to sport, splenic trauma, student athletes

## Abstract

Introduction: We aimed to evaluate the efficacy of measuring transaminase levels to determine the resolution of splenomegaly in athletes diagnosed with infectious mononucleosis (IM).

Methods: We collected serial aspartate aminotransferase (AST) and alanine transaminase (ALT) levels and ultrasound-measured spleen sizes in university athletes who had been diagnosed with IM. Our study included seven university-aged athletes from a single institution. Patients received serial liver function tests (LFT) and splenic ultrasound testing until resolution of symptoms and full return to sport. The effects of AST, ALT, and days from symptom onset were analyzed using multivariable mixed-effects linear regression models.

Results: Levels of AST and ALT were significantly correlated with spleen size. For each 10-unit increase in AST and ALT values, spleen size increased by 0.1 cm (p = 0.007) and 0.09 cm (p = 0.008), respectively. Decreasing levels of ALT and AST correlated with a decrease in spleen size. Normalization of AST/ALT values correlated with return of spleen size to baseline.

Conclusions: Liver function testing may be useful in the return-to-play decision-making process for athletes with IM.

## Introduction

Infectious mononucleosis (IM) is a widespread condition commonly caused by the Epstein-Barr virus (EBV). Splenomegaly is common in IM and a risk factor for splenic rupture [1]. Although splenic ultrasound (US) is considered the gold standard for determining splenomegaly, variability exists in baseline spleen sizes in athletes, and serial US may not be feasible in certain clinical settings. Because IM has a variable disease course, safe return to play (RTP) guidelines vary between individual athletes [2]. Effective monitoring of splenomegaly in athletes diagnosed with IM may prevent splenic rupture.

Splenic US is a rapid, non-invasive imaging modality that can be used to diagnose splenomegaly but requires adequate training of personnel to properly measure spleen size [3,4]. More objective measures of spleen size in the setting of IM could aid in guiding RTP. Previous studies have shown liver function tests (LFT) to be an effective predictor of acute IM with aspartate aminotransferase (AST) and alanine transaminase (ALT) elevation in nearly 100% of IM cases [5].

Much of the literature regarding splenic rupture in IM patients is in the form of case reports. In this retrospective study, we examine the correlation of transaminase levels with spleen size in IM. We hypothesize that transaminase measurements correlate with spleen size in patients with IM and serial LFT monitoring can help guide safe RTP in athletes after IM infection.

This article was previously presented as an abstract and poster at the IOC World Conference on Prevention of Injury and Illness in Sport between February 29 and March 2, 2024.

## Materials and methods

This study was performed as a retrospective review of patient data. Collegiate athletes at a single institution with a laboratory-confirmed diagnosis of IM and serial LFT and ultrasound measurements from 2/2020 through 2/2023 were included in this study. Individuals who were not varsity student-athletes at the participating university, those who did not have a diagnosis of IM confirmed with a monospot test, and/or were not monitored with serial ultrasound and LFT measurements during the period of their infection were excluded from this study. Serial AST and ALT levels and ultrasound-measured spleen sizes were collected post-IM diagnosis until resolution of symptoms and full return to sport. These results were monitored over an average course of 24 days (range: 18-33 days) post symptom onset.

The effects of AST, ALT, and days from symptom onset were analyzed using multivariable mixed-effects linear regression models. Analysis was done using RStudio version 2023.03.1+446 (Posit, Boston, MA) using a two-sided level of significance of 0.05. All analyses were performed by a biostatistician.

Anonymity of patient identity and secure handling of data were maintained throughout the analysis.

## Results

**Figure 1:**
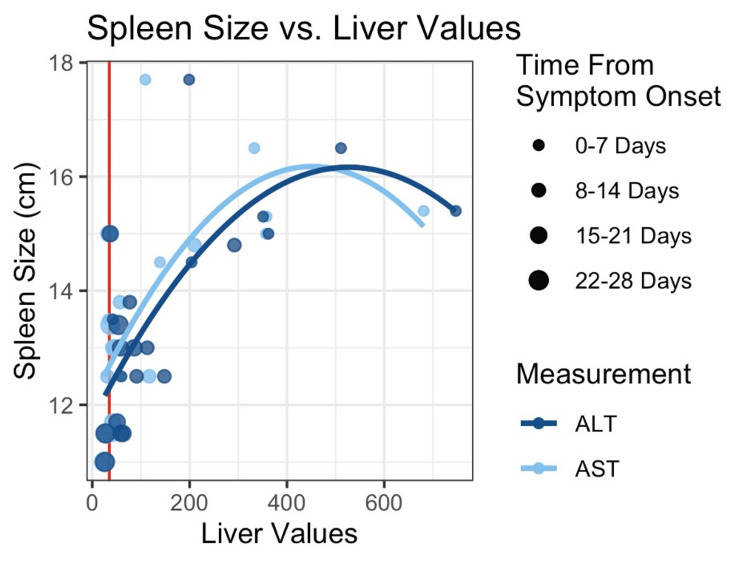
Spleen size vs. transaminase values. Spleen size was measured by ultrasound. Liver values include AST and ALT. AST: aspartate aminotransferase; ALT: alanine transaminase.

A total of five female and two male athletes (average age = 19.8 years, range = 17.8-21.2 years) competing in both contact and non-contact sports were included in the analysis. We observed a strong association between transaminase values and spleen size in our study population. Increased AST and increased ALT were significantly associated with increased spleen size, adjusting for the number of days from symptom onset (Figure 1). For each 10-unit increase in AST values, spleen size was expected to increase by 0.1 cm (p = 0.007). For each 10-unit increase in ALT values, spleen size was expected to increase by 0.09 cm (p = 0.008). As AST/ALT decreased from their peak, spleen size decreased as well.

**Figure 2:**
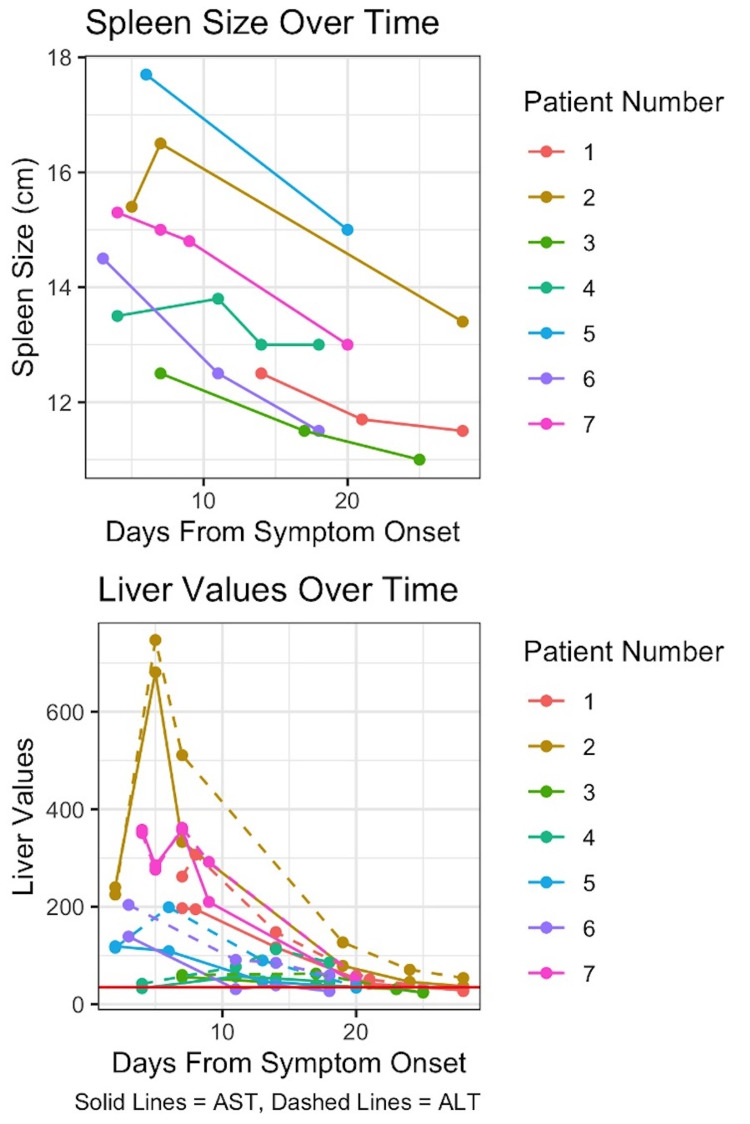
Correlation between transaminase values, spleen size, and days post symptom onset for individual athletes. AST: aspartate aminotransferase; ALT: alanine transaminase.

This strong correlation between transaminase values and spleen size was notable at the level of each individual athlete as well (Figure 2). The number of days since symptom onset was significantly associated with lower spleen size in both models, adjusting for transaminase values. For each additional day after symptom onset when adjusting for both AST and ALT, spleen size was expected to decrease by 0.08 cm (p = 0.008 and p = 0.004).

Our analysis demonstrated that ALT was a better predictor of spleen size than AST. Additionally, the quadratic terms for AST and ALT were negative and statistically significant. This indicates that as levels of AST and ALT increase, the effect on spleen size decreases.

## Discussion

Transaminase levels and spleen size are strongly correlated in athletes recovering from IM infection in this cohort of seven patients. This correlation could provide clinically relevant information for team physicians monitoring for resolution of splenomegaly in athletes diagnosed with IM. Because splenic rupture remains a central concern in the RTP decision-making process, the use of transaminase values to determine spleen size may offer a useful objective tool for athlete management when splenic US is not readily available or feasible. Although transaminases may be elevated due to causes other than IM, research by Wang et al. (2022) has shown that elevated transaminase values are highly specific to IM in this patient population [5].

The potential use of transaminase levels to evaluate spleen size and guide the RTP decision-making process is important as the use of ultrasound for this purpose remains controversial [2]. Notably, normal ranges of spleen size are largely based on Caucasian populations and do not necessarily represent normative spleen sizes in non-Caucasian patients [2]. Furthermore, baseline spleen size varies between individuals.

Although transaminase elevation can be observed due to non-infectious etiologies, Wang et al. (2022) demonstrated that in a cohort of 199 college athletes undergoing monospot testing, no patients were found to have elevated transaminases for reasons unrelated to IM [5]. This illustrates that transaminase levels provide sensitivity when evaluating college athletes for the severity of disease and are useful for the RTP decision-making process.

In their systematic review of published case reports including 85 patients with IM who experienced splenic rupture, Bartlett et al. (2016) found that the average time from onset of symptoms to splenic rupture was 14 days and that patients were at the highest risk in the first four weeks [6]. Our study monitored patients for an average of 28 days, which falls within this critical period of increased risk. By including data from this 28-day period, we feel confident that we have captured the most acute phase of splenomegaly associated with IM [6].

While our study cohort featured university-aged athletes, the use of transaminase values to evaluate splenic rupture risk likely has application in younger athletes. In their case report and RTP recommendations, Wolski et al. (2022) observed transaminitis in a pediatric IM patient who suffered splenic rupture at the age of 15 [7]. Their recommendation that pediatric patients are at highest risk during the first four weeks from symptom onset aligns with our study period. Similarly, Lee et al. (2020) observed transaminitis in a pediatric patient who suffered splenic rupture at the age of 13 [8]. The inclusion of younger pediatric patients with IM in future studies of splenic rupture is important as many children participate in sports and school-associated athletic programs.

Our study has several limitations. Our sample size was small with a cohort of seven collegiate student-athletes from a single American university. Future studies with larger cohorts may test our hypothesis with stronger statistical power and provide insight with further subgroup analyses. Future studies should also consider the enrollment of pediatric patients as well as patients across the country to determine the strength of association in a more diverse cohort. Additionally, institutional and provider differences in US technique may have an effect on reported US size. Furthermore, while our study followed patients for 28 days, cases of splenic rupture in IM patients have been reported up to eight weeks after symptom onset [6]. Though all transaminase values had normalized by the end of the monitoring period in this cohort, future studies should consider longer evaluation periods to capture cases of prolonged splenomegaly.

## Conclusions

The RTP decision-making process post IM infection is complex and goes beyond the risk of splenic rupture. Objective data help to quantify an athlete’s physiologic readiness to safely return to sport. Our study demonstrates that serial transaminase measurement may provide team physicians with a means to monitor spleen size in athletes who have been diagnosed with IM and help guide the RTP decision-making process. We found that both ALT and AST are strongly correlated with spleen size in each individual athlete in our study cohort. By utilizing these tests, athletes can be better informed in the RTP shared decision-making process, potentially reducing their risk of splenic rupture. Currently, case reports comprise much of the literature reporting on splenic rupture in patients with IM. While our study featured a small sample size, it serves as a pilot demonstrating the promise of LFTs, specifically transaminase levels, for determining splenomegaly in athletes with IM. This approach to IM management may also provide a useful solution in settings where ready access to ultrasound imaging is limited.
